# Feature of Heart Rate Variability and Metabolic Mechanism in Female College Students with Depression

**DOI:** 10.1155/2020/5246350

**Published:** 2020-02-27

**Authors:** Shanguang Zhao, Aiping Chi, Junhu Yan, Chong Yao

**Affiliations:** ^1^School of Sports, Shaanxi Normal University, Xi'an, China; ^2^Xuefu Hospital, Shaanxi Normal University, Xi'an, China; ^3^School of Psychology, Shaanxi Normal University, Xi'an, China

## Abstract

**Purpose:**

To explore the effects of depression on cardiac autonomic nerve function and related metabolic pathways, the heart rate variability (HRV) and urinary differential metabolites were detected on the college students with depression.

**Methods:**

12 female freshmen with depression were filtered by the Beck Depression Inventory (BDI-II) and Self-rating Depression Scale (SDS). By wearing an HRV monitoring system, time domain indexes and frequency domain indexes were measured over 24 hours. Liquid chromatography–mass spectrometry (LC-MS) was used to detect their urinary differential metabolites. Differential metabolites were identified by principal component analysis (PCA) and orthogonal projections to latent structures discriminant analysis (OPLS-DA). The metabolic pathways related to these differential metabolites were analyzed by the MetPA database.

**Results:**

Stress time was significantly increased, and recovery time was markedly decreased in the depression group compared with the control group (*p* < 0.001). Standard deviation of the normal-to-normal R interval (SDNN), root mean square of the beat-to-beat differences (RMSSD), high frequency (HF), and low frequency (LF) were decreased significantly (*p* < 0.001). Standard deviation of the normal-to-normal R interval (SDNN), root mean square of the beat-to-beat differences (RMSSD), high frequency (HF), and low frequency (LF) were decreased significantly (

**Conclusion:**

Some autonomic nervous system disruption, high stress, and poor fatigue recovery were confirmed in college students with depression. The metabolic mechanism involved the disruption of coenzyme Q biosynthesis, glycine-serine-threonine metabolism, tyrosine metabolism, pyrimidine metabolism, and steroid metabolism under daily stress.

## 1. Introduction

Depression is a severe mental illness with symptoms that include anxiety, insomnia, cognitive impairment, and even suicidal tendency [1, 2]. Studies investigating the causes and mechanisms of depression are research hotspots [3, 4]. The etiology and mechanism of depression are complex, and the biological abnormalities associated with depression involve many systems in the body, including the autonomic nervous system (ANS), which affects sleep. Therefore, somnipathy is also the most common clinical symptom of depression [5]. Heart rate variability (HRV), a reliable index that indicates stress level [6], can be used to quantitatively evaluate the tension and balance of the sympathetic nerve and vagus nerve in the heart [7]. HRV is widely used in many fields, such as clinical practice [8], sleep quality measurement [9], and stress and recovery analysis [10].

Metabonomics is a detection method used to analyze the metabolic mechanisms of an organism from the point view of molecular biology. This method can identify changes in differential metabolites and related metabolic pathways by detecting changes in small molecular metabolites [11]. Most modern theories of depression believe that stress leads to a risk of depression through cognitive processes and specific biological processes [12]. Some theories argue that major stressful events are one of the best predictors of depression [13]. Other studies have shown that certain life events, such as social exclusion, increase the risk of severe depression [14].

Adolescents are in the sensitive periods of physiological and psychological development, which means they lack of capability to handle learning stress, sleep lacking, interpersonal tension, and other external pressures and problems; thus, adolescents are prone to become depressed [15, 16]. The problem of depression among college students in China is highly prominent, with frequently occurred suicide caused by depression in recent years [17, 18]. College students with depression have symptoms such as insomnia and potentially increased duration of daily stress. However, it is not clear whether there is a direct relationship between daily stress time and autonomic nerve function, and the metabolic mechanism under stress remains unknown. Therefore, it is important to monitor college students with depression and explore their metabolic profiles.

In this study, 48 freshmen with depression were selected from Shaanxi Normal University by Beck Depression Inventory (BDI-II) and Self-rating Depression Scale (SDS). Their 24-hour dynamic HRV, pressure, and recovery time were monitored by a HRV monitoring system (Firstbeat Bodyguard 2). The urine samples were detected by differential metabolite detection, and related metabolic pathway analysis was performed to explore the metabolic mechanism.

## 2. Methods

### 2.1. Participants

A total of 4000 freshmen from Shaanxi Normal University were identified as having depression, according to the BDI-II and SDS, with the depression screening criteria of BDI-II score ≥ 15 [19] and SDS score ≥ 50 [20]. And then those students who meet the above conditions were required to pass a comprehensive physical examination at Xuefu Hospital of Shaanxi Normal University to eliminate other physical diseases, mental diseases, abnormal physical development (including), and other interference factors. Considering the sample size requirements of this experiment and excluding gender differences, 12 female students with depression were selected as the subjects of this study (depression group). Additionally, 12 healthy female students form the same grade were recruited as the control group (control group). The basic information of the subjects is shown in Table 1. All participants signed the informed consent. This study was approved by the Review Committee of Shaanxi Normal University.

### 2.2. Measurements and Analysis

To minimize the impact of external factors on HRV collection and differential metabolite detection, all participants were told to ban caffeine drinks, alcohol, tobacco, drugs, other foods, and strenuous exercise for 12 hours before monitoring.

HRV of the subjects was monitored by wearing an HRV monitoring system (Firstbeat Bodyguard 2, Finland) for 24 hours. The operation method: one electrode of the instrument was affixed to the right clavicle of the body, and the other was taped to the left rib of the body. The green LED light flashed when the device was successfully connected, and the data were automatically recorded. The instrument was removed when finished, and the HRV data were analyzed with linear (time and frequency domain) methods through the Firstbeat-compatible software. The time domain parameters were studied by the standard deviation of the artifact-eliminated (NN) intervals, the standard deviation of the normal-to-normal R interval (SDNN), and the root mean square of the beat-to-beat differences (RMSSD). SDNN reflects the overall HRV, and RMSSD is an indicator of cardiac parasympathetic regulation. Frequency bands in normal units (ms^2^) were obtained, including low frequency (LF) power (0.04 to 0.15 Hz) and high frequency (HF) power (0.15–0.4 Hz).

### 2.3. GC-MS Analysis of Urine Samples

Urine was selected as the sample for metabonomics analysis. All subjects were provided with the sane diet, and the morning urine in the middle of 2 mL was collected in a covered centrifuge tube, transported in a liquid nitrogen tank, and stored in a low temperature refrigerator at -80°C.

LC-MS analyses were performed using an UHPLC system (1290, Agilent Technologies) with a UPLC HSS T3 column (2.1 mm × 100 mm, 1.8 *μ*m) coupled to a Q Exactive mass spectrometer (Orbitrap MS, Thermo). The mobile phase A was 0.1% formic acid in water for positive and 5 mmol/L ammonium acetate in water for negative, and the mobile phase B was acetonitrile. The elution gradient was set as follows: 0 min, 1% B; 1 min, 1% B; 8 min, 99% B; 10 min, 99% B; 10.1 min, 1% B; and 12 min, 1% B. The flow rate was 0.5 mL/min. The injection volume was 1 *μ*L. The QE mass spectrometer was used due to its ability to acquire MS/MS spectra on an information-dependent basis (IDA) during an LC-MS experiment. In this mode, the acquisition software (Xcalibur 4.0.27, Thermo) continuously evaluates the full scan survey MS data as it collects and triggers the acquisition of MS/MS spectra depending on preselected criteria. The ESI source conditions were set as follows: sheath gas flow rate, 45 Arb; aux gas flow rate, 15 Arb; capillary temperature, 320°C; full MS resolution, 70,000; MS/MS resolution, 17,500; collision energy, 20/40/60 eV in the NCE model; and spray voltage, 3.8 kV (positive) or -3.1 kV (negative).

### 2.4. Statistical Analysis

In order to compare the age, weight, and BMI index, an independent sample *t*-test was carried out by the GraphPad Prism statistical software, and the results were expressed as mean ± standard deviation. The significance level among the groups was *p* < 0.05. To investigate the relationship between daily state and HRV parameters, multiple Pearson product-moment correlation coefficient (PPMCC) determinations were performed based on each outcome.

After LC-MS detection, the normalized data were analyzed by multivariate pattern recognition using the SIMCA software (V14, Umetrics AB, Umea, Sweden). Principal component analysis (PCA) of samples can reflect the overall differences among the samples and the degree of variation among the samples in the group as a whole. Using PCA and OPLS-PA multidimensional statistical processing, all compounds were screened for potential differential metabolites through the Kyoto Encyclopedia of Genes and Genomes (KEGG) database. The screening standards of differential metabolites were the variable importance in the projection (VIP) > 1, *p* < 0.05, and similarity > 700.

Finally, the screened differential metabolites were uploaded to the MetPA database (http://www.metaboanalyst.ca), and the influence weights of the corresponding metabolic pathways were analyzed. The influence score > 0.01 was used as the main criterion. The main metabolic pathways of college students with depression were identified.

## 3. Results

### 3.1. Stress and Recovery Time

**Figure 1:**
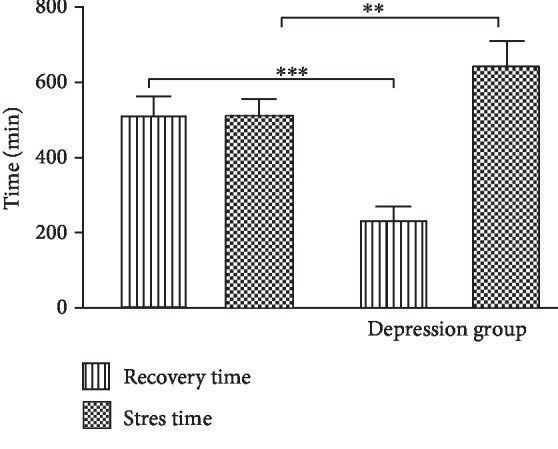
Change in the daily stress and recovery time of students with depression (^∗∗^*p* < 0.01, ^∗∗∗^*p* < 0.001).

Figure 1 presents the changes of the total stress and recovery time of students in two groups. The total stress time of students in the depression group was significantly increased in comparison with those in the control group (*p* < 0.01). However, the recovery time in the depression group was markedly decreased than that in the control group (*p* < 0.001). It was indicated that the daily stress management time was longer than the recovery time in students with depression.

### 3.2. HRV Data

The results of HRV data had statistical differences between the depression group and the control group. As shown in Table 2, RMSSD, R-R interval time, SDNN, HF, and LF of the depression group were significantly decreased (*p* < 0.05, 0.01, or 0.001) compared with those the control group, whereas the average heart rate (HR) was markedly elevated in the depression subjects (*p* < 0.01).

The results of correlation between the recovery time, stress time, and HRV parameters are presented in Table 3. The recovery time was negatively correlated with stress time and average HR and positively correlated with HF, RMSSD, and SDNN, and the results were significant or extremely significant (*p* < 0.05 or 0.01). However, the stress time was significantly negatively correlated with SDNN (*p* < 0.01).

### 3.3. LC-MS Detection Results of Urine Samples

**Figure 2:**
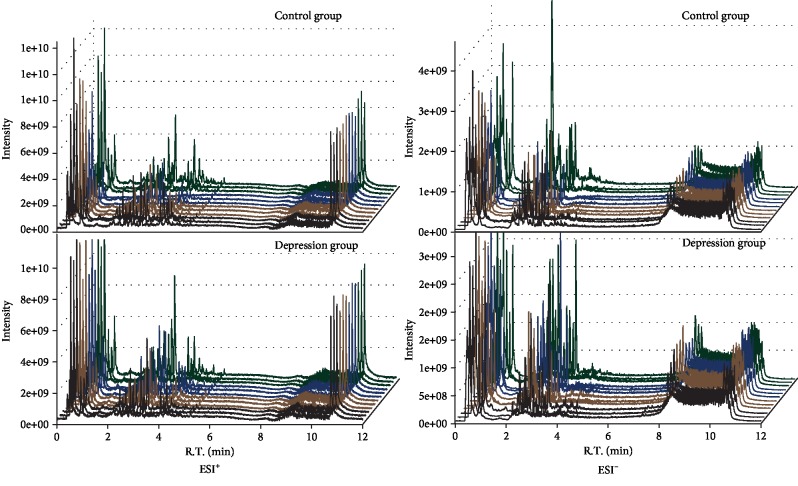
Total ion chromatograms (TICs) of urine samples from students (ESI+: positive ion model; ESI-: negative ion mode).

In this experiment, urine samples from the depression group and control group were collected to identify the differential metabolites between students with depression and healthy students. The results of the LC-MS ion flow diagram of urine samples are shown in Figure 2. Metabolomics was detected by LC-MS. The original mass spectrometry peaks obtained from 24 samples were normalized, and the peak area was calculated by the SIMCA software.

### 3.4. Multidimensional Statistical Analysis of the Differential Metabolites

**Figure 3:**
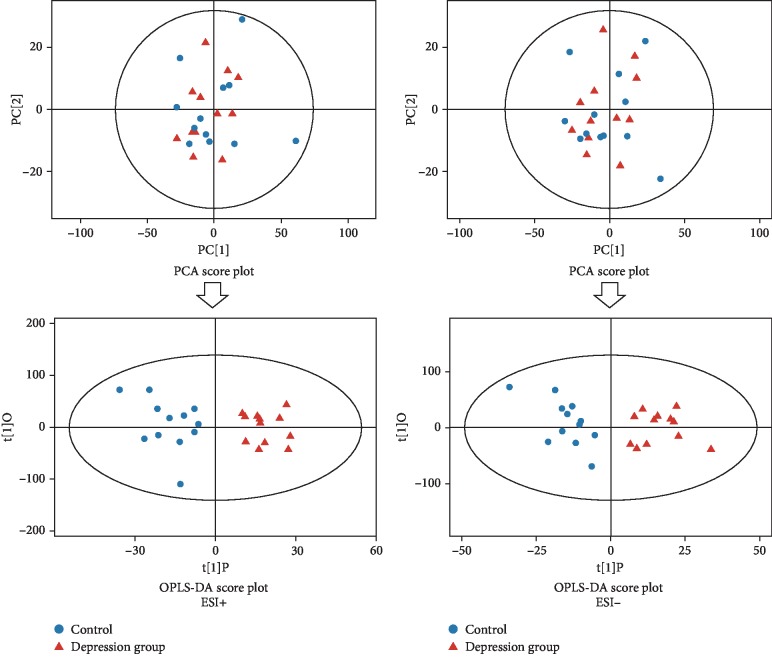
PCA score plot and OPLS-DA score plot of urine sample from the subjects in the depression and control groups (ESI+: positive ion model; ESI-: negative ion mode).

The peak area data from mass spectrometry were analyzed by multivariate pattern recognition; first, PCA [21] was performed. PCA is a statistical method that converts a group of observed possible correlation variables into linear uncorrelated variables (principal components) through orthogonal transformation. PCA can reveal the internal structure of the data, thus facilitating better interpretation of data variables. Due to the influence of related variables, different variables will be scattered across more principal components, preventing perform good visualization and follow-up analysis. Therefore, we used the statistical method of orthogonal projections to latent structures-discriminant analysis (OPLS-DA) to analyze the results. Through OPLS-DA analysis, the orthogonal variables that were not related to the classification variables were filtered out, and the nonorthogonal variables and orthogonal variables were analyzed. To obtain reliable information about the differences in metabolites between groups and the degree of correlation between the depression group and the control group the PCA and OPLS-DA, results of the urinary metabolites between the depression group and control group are shown in Figure 3.

Each point in the diagram represents a sample, and the coordinates of the sample in the diagram were determined by the composition it contains; in other words, the difference in the distribution of the sample was determined by differences in composition. The samples from the depression group and the control group were distributed in each quadrant, which showed that PCA analysis alone rarely achieved an obvious separation effect, so it was necessary to further analyze the samples with supervised OPLS-DA. The unrelated orthogonal signals were filtered out by OPLS-DA, making the obtained differential metabolites more reliable. From the OPLS-DA score diagram, the control group samples were distributed in the second and third quadrants, while the depression group samples were distributed in the first and fourth quadrants; the distribution of the two groups of samples was very neat, and the separation effect was very obvious. The OPLS-DA score reflected the similarity of urines within the groups and the differences in urine metabolites between the depression group and the control group.

### 3.5. Screening of Potentially Differential Metabolites

To filter out irrelevant orthogonal signals and to obtain more reliable differential metabolites, not only must the VIP value of a metabolite exceed 1 in the OPLS-DA model but also its *p* value must be less than 0.05 between groups in Student's *t*-test. KEGG mass spectral libraries were utilized to ensure the identification accuracy of the differential metabolites, and then the similarity value of the compounds needed to exceed 700 to be adopted.

After the above filtrations, the 15 metabolites were identified in the depression group in comparison with the control group as follows: methanoic acid, glycine ursodeoxycholic acid, 3-hydroxyshiptic acid, m-cresol, 4-hydroxyphenyl lactic acid, azelaic acid, vanillin, dimethyl glycine, gentian acid, ethylene glycol, dihydrothymine, corticosterone, indole methanol, methyl uridine, and p-ethyl benzoic acid. In the negative ion mode, the 10 substances that follow were identified: methanoic acid, glycine ursodeoxycholic acid, 3-hydroxyshiptic acid, m-cresol, 4-hydroxyphenyl lactic acid, azelaic acid, vanillin, dimethyl glycine, gentian acid, and ethylene glycol. Five of these were also identified in positive ion mode, namely, dihydrothymine, corticosterone, indole methanol, methyl uridine, and p-ethyl benzoic acid (Table 4).

The relative values of the peak areas of the differential metabolites are shown in Table 4. The results demonstrated that the values of malonic acid, fumaric acid, 2-methylfumarate, L-malic acid, and palmitic acid were significantly increased, whereas the values of 4-acetamidobutyric acid, *α*-ketoglutaric acid, tartaric acid, gluconic acid, sphingosine, and 21-hydroxypregnenolone were markedly decreased, except for the significant increase in methyl uridine and in the depression group compared with the control group in positive ion mode. In negative ion mode, the values of m-cresol, vanillin, and dimethyl glycine were significantly increased, while the values of methanoic acid, glycine ursodeoxycholic acid, 3-hydroxyshiptic acid, 4-hydroxyphenyl lactic acid, azelaic acid, and gentian acid ethylene glycol were markedly decreased.

### 3.6. Attribution of Metabolic Pathways

**Figure 4:**
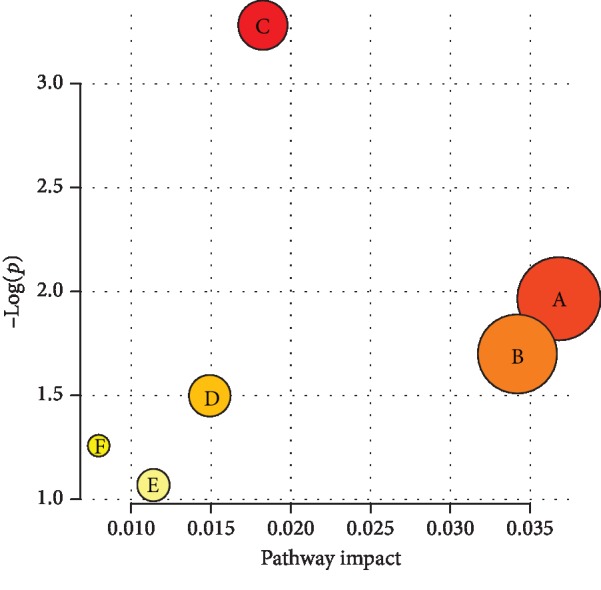
Results of the metabolic pathway topology analysis. (a: coenzyme Q biosynthesis; b: glycine-serine-threonine metabolism; c: tyrosine metabolism; d: pyrimidine metabolism; e: steroid biosynthesis; f: tryptophan metabolism).

Although there are more differential metabolites than those mentioned above, it is necessary to further evaluate the weight and influence of the metabolic pathway and its related metabolic pathways. At present, the accepted metabolic pathway evaluation method is MetaboAnalyst (http://www.metaboanalyst.ca/). Characterization analysis, hypergeometric test, path topological structure analysis, relative centrality selection, and topological weight score analysis were performed on MetaboAnalyst. The results of the main identified metabolic pathways with changes were shown in Figure 4. Five metabolic pathways were generated in the depression group in comparison with the control groups, and the pathway impact values of those metabolic pathways were calculated via a pathway topology analysis with a threshold of 0.01. These pathways were coenzyme Q biosynthesis, glycine-serine-threonine metabolism, tyrosine metabolism, pyrimidine metabolism, and steroid biosynthesis. The tryptophan metabolic pathway was excluded because the impact values did not meet the standard. The results suggested that there were characteristic disorders in the five above metabolic pathways in students with depression.

## 4. Discussion

In this study, the time domain indexes RMSSD and SDNN and the frequency domain indexes LF and HF were selected. The results showed that the overall level of HRV of college students with depression was lower than that of healthy control students, which is in accordance with the results of recent studies on depression [22–26]. In terms of the time domain indexes, the SDNN and RMSSD were lower in the depression group than in the control group [23–25]; in terms of the frequency domain indexes, the LF and HF were much lower in the depression group than in the control group [27, 28]. The results of the Firstbeat test showed that the daily stress time of college students with depression was significantly longer than that of healthy college students, while both the recovery time and sleep time are significantly shorter of college students with depression than those of healthy college students. Stress occurs after 11 : 00 in the evening, leading to sleep disorders in college students with depression [29]. In addition, the potential neurophysiological mechanisms of stress and recovery time were studied by analyzing HRV parameters. The significant results show that stress time may be the factor that leads to the decrease in HRV parameters, and the recovery time may be the potential factor for the recovery of these HRV parameters. The longer the recovery time is, the more helpful it is to the balance of the ANS and to reduce depression. The SDNN in the time domain index reflects the control ability and recovery degree of the autonomic nerve to the heart. The RMSSD is a sensitive index that reflects the vagus nerve [30], which means that the larger the value is, the stronger the autonomic nerve regulation ability. HF power is an index that reflects the change in vagus nerve activity, and an increase in HF power indicates that the activity of the vagus nerve is enhanced [31]. LF power is a compound regulatory function of the sympathetic nerve and vagus nerve, which further reflects the heart rate changes caused by baroreceptor reflex and blood pressure regulation [32]. Some research reported that HF and LF were significantly reduced in patients with chronic fatigue syndrome (CFS) or anxiety, which discovered there were an imbalance of cardiac autonomic nerves and the decreased cardiac vagus function in some mental illness [33, 34]. Moreover, a recent study reported the average heart rate was increased in patients with depression [35]. In the present study, our results demonstrated that both the HF and LF of the subjects with depression were significantly reduced, and the average heart rate was significantly increased, confirming that depression could also cause an imbalance of cardiac autonomic nerves and the decreased cardiac vagus function in college students. In summary, compared with healthy college students, students with depression have longer stress time and shorter recovery time. In addition, the results of the HRV experiment in this study show that the cardiac autonomic nerve function of college students with depression is disrupted, which affects sleep quality and the mental state. The above factors are intrinsically related because a lack of sleep leads to a reduction in recovery time, and both stress and lack of sleep will further lead to of the disrupted autonomic nerve function in college students with depression.

The current research, including the 5-HT hypothesis, dopamine hypothesis, amino acid neurotransmitter hypothesis, norepinephrine hypothesis, acetylcholine hypothesis, neuroendocrine function changes, and neuroimmune theory [36, 37], believes that the pathogenic factors and pathogenesis of depression are mainly understood from the three major systems, the nervous, endocrine, and immune systems. There are some differences in the metabolic mechanism of depression. Pan et al. and Moaddel et al. [38, 39] detected differential plasma metabolites in patients with depression through LC-MS and showed that differential characteristic plasma metabolites were mainly concentrated in lipid metabolic pathways (such as LDL, VLDL, unsaturated lipids, and cholesterol), energy metabolic pathways (such as glucose, pyruvate, and lactic acid), and amino acid metabolic pathways (such as alanine, glycine, and taurine). Vahabi et al. [40] detected serum samples of patients with depression with ^1^HNMR and found that the levels of the differential metabolites of some amino acids (glutamic acid, glutamine, alanine, N-acetyl glycoprotein, leucine, and isoleucine) in serum were significantly increased. Ratnasekhar et al. detected urine samples from patients with depression by GC-MS, and the results showed that the urine markers were azelaic acid, sorbitol, uric acid, quinolinic acid, hippuric acid, and tyrosine [41]. Goedert et al. used ^1^HNMR to detect fecal samples from patients with depression. The results revealed that the metabolic mechanism of depression is related to the disruption of lipid metabolism and amino acid metabolism [42]. The similarities and differences of the above studies are due to the different detection methods used and the different samples collected. In this research, LC-MS was used to detect urine samples from students with depression. Our results showed that 15 characteristic differential metabolites are present in patients with depression. The relative contents of methyl uridine, m-cresol, vanillin, and dimethyl glycine were significantly increased, while the relative contents of 11 additional metabolites, including dihydrothymine, corticosterone, indole methanol, 4-ethylbenzoic acid, methanoic acid, glycoursodeoxycholic acid, 3-hydroxyshiptic acid, 4-hydroxyphenyl lactic acid, azelaic acid, 2,5-dihydroxybenzoic acid, and ethylene glycol were significantly decreased in the depression students when compared with the healthy students. The metabolic pathways related to the differential metabolites in the college students with depression mainly included coenzyme Q biosynthesis, glycine-serine-threonine metabolism, tyrosine metabolism, glycine-serine-threonine metabolism, pyrimidine metabolism, and steroid hormone biosynthesis.

The results of glycine-serine-threonine metabolism and the tyrosine metabolism pathway are consistent with the results of other studies investigating depression by Pan et al., Moaddel et al., and Ratnasekhar et al. [38, 39, 41]. Corticosterone is a key substance in the steroid hormone biosynthesis pathway. Prior studies showed that the severity of depression was negatively correlated with corticosterone levels [43, 44]. Our study also confirmed a significant decrease in corticosterone in students with depression; meanwhile, the steroid hormone biosynthesis pathways have been detected.

Moreover, this experiment found that the coenzyme Q biosynthesis pathway is disrupted in students with depression. Coenzyme Q has many physiological functions, such as reducing the production of free radicals in the myocardium and skeletal muscle and enhancing the physical strength of patients with heart disease [45]. Other research has shown that the synthesis of coenzyme Q10 affects the function of mitochondria, resulting in depression [46]. Therefore, some studies suggested that coenzyme Q10 may be a new target for the treatment of depression [47, 48]. Our results show that the relative level of 4-hydroxyphenyl lactic acid, the key metabolite in the coenzyme Q biosynthesis pathway, decreased significantly in the depression group, which suggests that metabolic problems in coenzyme Q biosynthesis may contribute to this process.

In addition, this study found that the relative level of dihydrothymine in the pyrimidine metabolic pathway was significantly decreased in students with depression. Dihydrothymine is a marker of thymine synthesis and metabolism that is often used to evaluate the metabolic level of thymine [49]. As a basic base unit of DNA and RNA, thymine plays an important physiological role in skin cancer caused by ultraviolet injury and in other diseases [50]. The results suggest that the level of thymine metabolism in students with depression is lower than that in healthy students, which could be related to the myocardial fatigue caused by insufficient sleep. Moreover, college students with depression have a serious lack of sleep, and their time under stress is longer than their recovery time, which leads to the disruption of the pyrimidine metabolic pathway.

## 5. Conclusions

The overall level of daily HRV in college students with depression is lower than that in healthy college students. The daily stress time is greater than the recovery time, which could be one of the main causes of autonomic dysfunction. Metabonomic tests showed that disorders exist in five metabolic pathways, including coenzyme Q biosynthesis, glycine-serine-threonine metabolism, tyrosine metabolism, pyrimidine metabolism, and steroid hormone biosynthesis, in college students with depression.

## Figures and Tables

**Table 1:** Basic information of the subjects.

Groups	Age	Height	Weight	BMI^a^
Control group (*n* = 12)	18-20	161.5 ± 5.9	51.39 ± 2.1	20.31 ± 2.1
Depression group (*n* = 12)	18-20	160.7 ± 6.7	50.00 ± 1.9	19.4 ± 1.6

^a^Body mass index.

**Table 2:** Comparative results for the HRV parameters in the depression group *vs*. control group.

	HR	R-R (ms)	RMSSD (ms)	SDNN (ms)	HF (ms^2^)	LF (ms^2^)
Control group (*n* = 12)	74.40 ± 3.31	18.63 ± 1.15	60.50 ± 10.54	182.25 ± 37.42	1180.74 ± 269.62	1582.00 ± 40.52
Depression group (*n* = 12)	83.22 ± 8.04^∗^	17.11±0.92^∗∗^	33.78±12.69^∗∗∗^	130.00±35.05^∗∗^	931.31±363.03^∗∗^	967.61±196.19^∗∗∗^

Note: *vs*. control group—^∗^*p* < 0.05, ^∗∗^*p* < 0.01, and ^∗∗∗^*p* < 0.001.

**Table 3:** Correlation analysis of the recovery time, stress time, and HRV parameters.

		Recovery time	Stress time	HR	R-R	RMSSD (ms)	SDNN (ms)	HF (ms^2^)	LF (ms^2^)
Recovery time	*p*	1	-0.542^∗^	-0.740^∗∗^	0.368	0.727^∗∗^	0.648^∗∗^	0.602^∗^	0.480
	*r*		0.025	0.001	0.146	0.001	0.005	0.011	0.051

Stress time	*p*	-0.542^∗^	1	0.147	-0.374	-0.353	-0.699^∗∗^	-0.471	-0.481
	*r*	0.025		0.573	0.139	0.165	0.002	0.056	0.051

Note: ^∗^*p* < 0.05 and ^∗∗^*p* < 0.01.

**Table 4:** Potential differential metabolites in the depression group in comparison with the control group.

	Number	Metabolite	HMDB ID	KEGG ID	RT (min)	*m*/*z*	Similarity	VIP	*p* value	Trend
ESI^+^	1	Dihydrothymine	HMDB0000079	C00906	115.58	129.06	0.741	2.31	0.017	↓^∗^
	2	Cortisone	HMDB0002802	C00762	280.76	361.20	0.711	2.21	0.022	↓^∗^
	3	Indole-3-carbinol	HMDB0005785	—	213.40	147.04	0.686	2.49	0.004	↓^∗∗^
	4	4-Ethylbenzoic acid	HMDB0002097	—	248.36	151.07	0.537	1.84	0.038	↓^∗^
	5	3-Methyluridine	HMDB0004813	—	55.189	259.09	0.638	1.77	0.044	↑^∗^

ESI^−^	1	Formylanthranilic acid	HMDB0004089	C05653	145.02	164.03	0.974	1.98	0.007	↓^∗∗^
	2	Glycoursodeoxycholic acid	HMDB0000708	—	271.957	448.31	0.937	1.88	0.038	↓^∗^
	3	3-Hydroxyhippuric acid	HMDB0006116	—	133.45	194.05	0.898	2.33	0.013	↓^∗^
	4	m-Cresol	HMDB0002048	C01467	68.77	107.05	0.828	2.38	0.011	↑^∗^
	5	Hydroxyphenyllactic acid	HMDB0000755	C03672	69.68	181.05	0.808	2.01	0.012	↓^∗^
	6	Azelaic acid	HMDB0000784	C08261	109.58	187.09	0.778	1.822	0.031	↓^∗^
	7	Vanillin	HMDB0012308	C00755	68.72	151.04	0.768	2.47	0.010	↑^∗^
	8	Dimethylglycine	HMDB0000092	C01026	32.81	102.06	0.726	2.93	0.012	↑^∗^
	9	Gentisic acid	HMDB0000152	C00628	76.71	153.02	0.718	1.99	0.049	↓^∗^
	10	17a-Ethynylestradiol	HMDB0001926	C07534	229.01	295.17	0.539	1.33	0.049	↓^∗^

Note: *vs*. control group—^∗^*p* < 0.05 and ^∗∗^*p* < 0.01.

## Data Availability

All data generated or used during the study appear in the submitted article. Some raw data generated or used during the study are available from the corresponding author by request.
